# Supplementary material for: Metabolic Signature Differentiated Diabetes Mellitus from Lipid Disorder in Elderly Taiwanese
Source: J Clin Med. 2018 Dec 21;8(1):13. doi: 10.3390/jcm8010013 (PMC6352219; doi:10.3390/jcm8010013)
Supplement: Supplementary file 1 [file jcm-08-00013-s001.pdf]

Supplementary Materials

Table S1. The integral for 3-(Trimethylsilyl) propionic-2,2,3,3-d4 acid (TSP) in each daily quality control sample.

Icon	QC Name	Integral for TSP
411	CGHACV-HPmix-0527	253550861.9
1002	CGHACV-HPmix-0528	260904230.4
1451	CGHACV-HPmix-0528	258399212.8
1461	CGHACV-HPmix-0527	252098438.5
2002	CGHACV-HPmix-0528	256457360.6
2491	CGHACV-HPmix-0529	266639207.8
3002	CGHACV-HPmix-0601	271227629.1
3511	CGHACV-HPmix-0601	260588700.1
4002	CGHACV-HPmix-0602	265023189.3
4011	CGHACV-HPmix-0601	258862402
4501	CGHACV-HPmix-0602	255274986.1
5002	CGHACV-HPmix-0603	275669920
5011	CGHACV-HPmix-0602	261232265.9
5501	CGHACV-HPmix-0603	265771864
6002	CGHACV-HPmix-0604	259300243.3
6501	CGHACV-HPmix-0604	256783949.9
7002	CGHACV-HPmix-0605	283994756.1
7011	CGHACV-HPmix-0604	262606682.3
7501	CGHACV-HPmix-0605	257597022.6
8002	CGHACV-HPmix-0608	263263692.5
8503	CGHACV-HPmix-0608	284468604.5
9002	CGHACV-HPmix-0610	277178778
9011	CGHACV-HPmix-0608	271200908.8
9411	CGHACV-HPmix-0610	273840264.3
Mean ± SD	264663965.5 ± 9132478.287	
CV	3.50%	

Table S2. Metabolites of plasma were significantly different between lipid disorder (LD) and control groups.

Metabolite	Chemical Shift (ppm)	Control (n = 47)	Lipid Disorder (n = 43)	q-Value
Phenylalanine	7.420	0.011487 ± 0.001401	0.012949 ± 0.002050	0.0032
Tyrosine	7.189	0.019911 ± 0.002389	0.021267 ± 0.002784	0.1726
Histidine	7.053	0.011308 ± 0.001238	0.012009 ± 0.001322	0.0623
Urea	5.743	0.025949 ± 0.007283	0.034424 ± 0.018936	0.0441

Lipid (CH=CH)	5.310	0.631180 ± 0.134659	1.010308 ± 0.303849	<0.001
◎ -Glucose	5.230	0.206520 ± 0.024224	0.257082 ± 0.057782	<0.001
Lactate	4.105	0.179851 ± 0.032207	0.233029 ± 0.046372	<0.001
Citrate	2.647	0.090115 ± 0.008446	0.099493 ± 0.010397	<0.001
Pyruvate	2.364	0.033364 ± 0.006008	0.042581 ± 0.008158	<0.001
Lipid (CH2-CO)	2.220	0.090617 ± 0.040856	0.254925 ± 0.116124	<0.001
Lipid (CH2-CH=CH)	2.005	1.143546 ± 0.180516	1.666382 ± 0.353365	<0.001
Lipid (CH2-CH2-CO)	1.561	0.219842 ± 0.084980	0.545055 ± 0.220494	<0.001
Alanine	1.473	0.139857 ± 0.021317	0.173221 ± 0.031349	<0.001
LDL	1.245	3.309798 ± 0.909422	6.247461 ± 2.081391	<0.001
Valine	1.035	0.068262 ± 0.009896	0.084637 ± 0.012807	<0.001
Isoleucine	1.000	0.032724 ± 0.005215	0.046973 ± 0.009015	<0.001
Leucine	0.954	0.138770 ± 0.016923	0.187542 ± 0.034333	<0.001
VLDL	0.870	1.331665 ± 0.309550	2.124258 ± 0.603458	<0.001

Data are mean ± SD. Generalized linear models were used to analyze the effect of variable controlling for age and comorbidities, including hypertension, coronary artery disease (CAD), stroke, chronic kidney disease (CKD). Model significance was presented as adjusted *p*-value. The false discovery rate (FDR) was applied to the *q*-values obtained from the tests.

Table S3. The concentration of metabolites were significantly different between lipid disorder (LD) and control (Con) groups.

Metabolites (μM)	Con (n = 40)	Con + DM (n = 3)	LD (n = 20)	LD+DM (n = 19)	One-Way ANOVA	Adjusted p-Value (Con/LD)	q-Value (Con/LD)	VIP
PC ae C34:3	8.27 ± 1.87	10.17 ± 2.21	5.67 ± 1.36	4.81 ± 0.88	<0.001	<0.001	<0.001	1.79
Glutamate	39.93 ± 13.48	32.03 ± 11.15	68.81 ± 23.46	80.82 ± 24.25	<0.001	<0.001	<0.001	1.75
PC ae C44:6	1.64 ± 0.42	1.58 ± 0.37	1.17 ± 0.30	1.07 ± 0.30	<0.001	<0.001	<0.001	1.55
PC ae C42:4	0.88 ± 0.17	0.78 ± 0.20	0.68 ± 0.18	0.61 ± 0.15	<0.001	<0.001	<0.001	1.50
PC ae C32:2	0.60 ± 0.10	0.72 ± 0.23	0.46 ± 0.10	0.39 ± 0.10	<0.001	<0.001	<0.001	1.65
Alanine	322.38 ± 77.45	313.67 ± 9.07	449.50 ± 85.51	505.63 ± 101.78	<0.001	<0.001	<0.001	1.75
PC ae C44:3	0.12 ± 0.02	0.13 ± 0.02	0.10 ± 0.02	0.10 ± 0.02	<0.001	<0.001	<0.001	1.46
PC ae C44:4	0.34 ± 0.07	0.31 ± 0.06	0.26 ± 0.06	0.25 ± 0.05	<0.001	<0.001	<0.001	1.45
PC aa C42:0	0.72 ± 0.18	0.74 ± 0.19	0.53 ± 0.14	0.49 ± 0.16	<0.001	<0.001	<0.001	1.45
PC aa C42:2	0.38 ± 0.08	0.42 ± 0.13	0.30 ± 0.06	0.29 ± 0.08	<0.001	<0.001	<0.001	1.30
PC ae C34:2	10.34 ± 1.87	11.83 ± 1.60	8.63 ± 2.46	6.74 ± 1.28	<0.001	<0.001	0.0014	1.58
PC ae C42:3	0.87 ± 0.16	0.97 ± 0.21	0.70 ± 0.15	0.66 ± 0.17	<0.001	<0.001	0.0015	1.37
PC aa C38:3	38.44 ± 10.23	47.83 ± 18.03	50.75 ± 9.67	43.94 ± 9.12	<0.001	<0.001	0.0015	1.49
PC ae C36:3	6.68 ± 1.09	7.60 ± 0.78	5.67 ± 1.50	4.49 ± 0.94	<0.001	<0.001	0.0018	1.57
PC ae C38:2	1.65 ± 0.30	1.79 ± 0.29	1.38 ± 0.31	1.19 ± 0.29	<0.001	<0.001	0.0030	1.39
alpha-AAA	0.98 ± 0.17	0.89 ± 0.08	1.28 ± 0.30	1.38 ± 0.37	<0.001	<0.001	0.0033	1.46
PC ae C44:5	1.42 ± 0.36	1.34 ± 0.23	1.12 ± 0.33	0.96 ± 0.26	<0.001	<0.001	0.0038	1.30
PC ae C32:1	2.25 ± 0.43	2.47 ± 0.62	1.86 ± 0.49	1.54 ± 0.36	<0.001	<0.001	0.0038	1.43
PC ae C42:5	1.75 ± 0.32	1.77 ± 0.20	1.48 ± 0.32	1.27 ± 0.25	<0.001	<0.001	0.0039	1.38
PC aa C42:1	0.42 ± 0.11	0.44 ± 0.14	0.32 ± 0.09	0.31 ± 0.10	<0.001	<0.001	0.0039	1.24
PC aa C40:2	0.56 ± 0.21	0.61 ± 0.14	0.44 ± 0.08	0.41 ± 0.10	<0.001	0.0012	0.0073	1.02
PC aa C40:1	0.58 ± 0.12	0.64 ± 0.16	0.49 ± 0.10	0.47 ± 0.12	<0.001	0.0010	0.0075	1.04
Valine	203.10 ± 27.03	206.33 ± 7.57	232.85 ± 23.39	244.84 ± 26.91	<0.001	0.0012	0.0076	1.48
PC ae C40:4	2.07 ± 0.37	2.25 ± 0.34	1.82 ± 0.32	1.59 ± 0.30	<0.001	0.0012	0.0078	1.31
PC ae C40:3	1.27 ± 0.17	1.37 ± 0.19	1.12 ± 0.18	0.98 ± 0.20	<0.001	0.0014	0.0078	1.40
PC aa C36:4	113.70 ± 11.56	127.67 ± 11.59	127.10 ± 11.89	118.00 ± 13.17	0.0049	0.0013	0.0078	1.48
PC aa C36:3	86.97 ± 9.43	100.43 ± 12.62	97.75 ± 8.70	92.05 ± 10.05	0.0047	0.0011	0.0079	1.45
SM C16:0	217.03 ± 27.90	226.67 ± 23.35	193.65 ± 37.90	165.68 ± 30.68	<0.001	0.0016	0.0085	1.42
PC aa C34:1	109.40 ± 10.58	121.00 ± 15.62	121.50 ± 12.58	116.03 ± 13.18	0.0060	0.0017	0.0090	1.28
PC aa C40:3	0.79 ± 0.25	0.82 ± 0.11	0.65 ± 0.12	0.59 ± 0.17	<0.001	0.0021	0.0105	1.04

lysoPC a C18:2	40.64 ± 14.69	36.37 ± 9.36	30.92 ± 7.49	32.32 ± 7.48	0.0039	0.0021	0.0107	1.02
PC ae C38:1	0.33 ± 0.14	0.44 ± 0.11	0.22 ± 0.15	0.17 ± 0.11	<0.001	0.0031	0.0151	1.18
Glycine	297.70 ± 61.10	373.00 ± 127.08	253.15 ± 49.19	253.68 ± 45.92	0.0023	0.0035	0.0164	0.96
AC C3	0.34 ± 0.13	0.32 ± 0.21	0.50 ± 0.20	0.52 ± 0.16	0.0023	0.0041	0.0185	1.24
Leucine	126.87 ± 20.76	122.33 ± 9.71	149.90 ± 18.94	162.93 ± 26.89	<0.001	0.0044	0.0189	1.48
PC ae C42:2	0.57 ± 0.11	0.67 ± 0.15	0.49 ± 0.10	0.45 ± 0.13	<0.001	0.0044	0.0192	1.09
PC aa C34:2	106.81 ± 9.77	115.33 ± 10.12	116.35 ± 10.99	111.89 ± 11.43	0.0165	0.0046	0.0193	1.17
PC ae C40:5	3.33 ± 0.64	3.77 ± 0.47	2.92 ± 0.51	2.58 ± 0.58	<0.001	0.0050	0.0203	1.22
PC ae C36:2	11.79 ± 1.83	13.27 ± 2.06	10.62 ± 1.87	9.32 ± 1.91	<0.001	0.0057	0.0225	1.25
PC ae C40:2	1.78 ± 0.40	2.05 ± 0.49	1.51 ± 0.28	1.31 ± 0.39	<0.001	0.0073	0.0279	1.22
PC aa C36:2	99.13 ± 9.28	107.33 ± 7.51	107.94 ± 10.40	103.65 ± 11.40	0.0284	0.0081	0.0298	1.13
SM C24:1	231.08 ± 35.45	236.33 ± 19.86	201.30 ± 52.80	162.63 ± 37.87	<0.001	0.0080	0.0301	1.43
AC C14:1	0.09 ± 0.02	0.10 ± 0.02	0.08 ± 0.01	0.07 ± 0.02	0.0017	0.0086	0.0302	1.08
AC C5	0.08 ± 0.02	0.07 ± 0.02	0.10 ± 0.04	0.11 ± 0.04	0.0087	0.0085	0.0303	1.16
AC C4	0.24 ± 0.07	0.17 ± 0.03	0.32 ± 0.15	0.31 ± 0.10	0.0307	0.0117	0.0401	1.03
SM C26:1	0.96 ± 0.23	1.04 ± 0.30	0.82 ± 0.27	0.63 ± 0.15	<0.001	0.0134	0.0447	1.33
Phenylalanine	75.05 ± 7.51	71.60 ± 9.97	83.63 ± 9.21	83.76 ± 11.64	0.0113	0.0148	0.0485	1.15

Data are mean ± SD. Variables were analyzed by one-way analysis of variance (ANOVA) between four groups, and independent sample *t*-tests were used to compare the control and LD groups. Generalized linear models were used to analyze the effect of variables controlling for age and comorbidities, including hypertension, coronary artery disease (CAD), stroke, chronic kidney disease (CKD). Model significance was presented as adjusted *p*-value. The false discovery rate (FDR) was applied to the *q*-values obtained from the tests. DM, diabetes mellitus; alpha-AAA, alpha-aminoadipic acid; lysoPC a, lysophosphatidylcholine acyl; PC aa, phosphatidylcholine diacyl; PC ae, phosphatidylcholine acyl-alkyl; SM, sphingomyelin; AC, acylcarnitine.

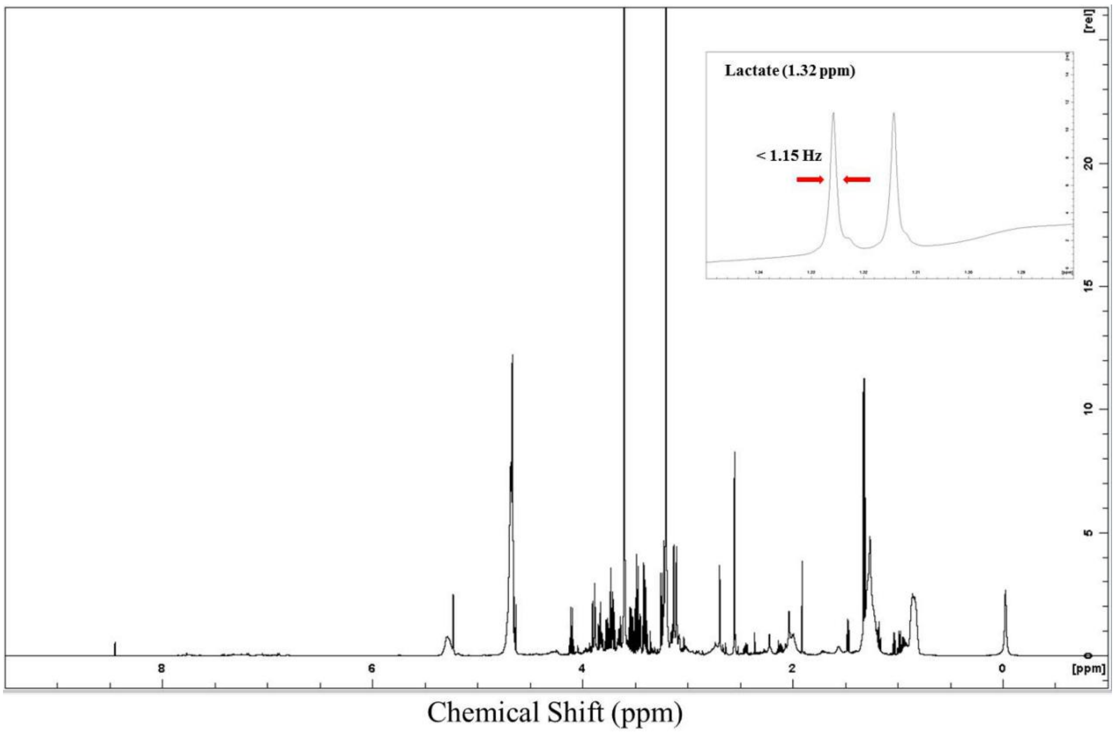

Figure S1. 1D ^{1}H NMR spectrum of quality control (human plasma). The line width at half height is <1.15 Hz for one of the lactate resonances at a chemical shift of 1.32 ppm.

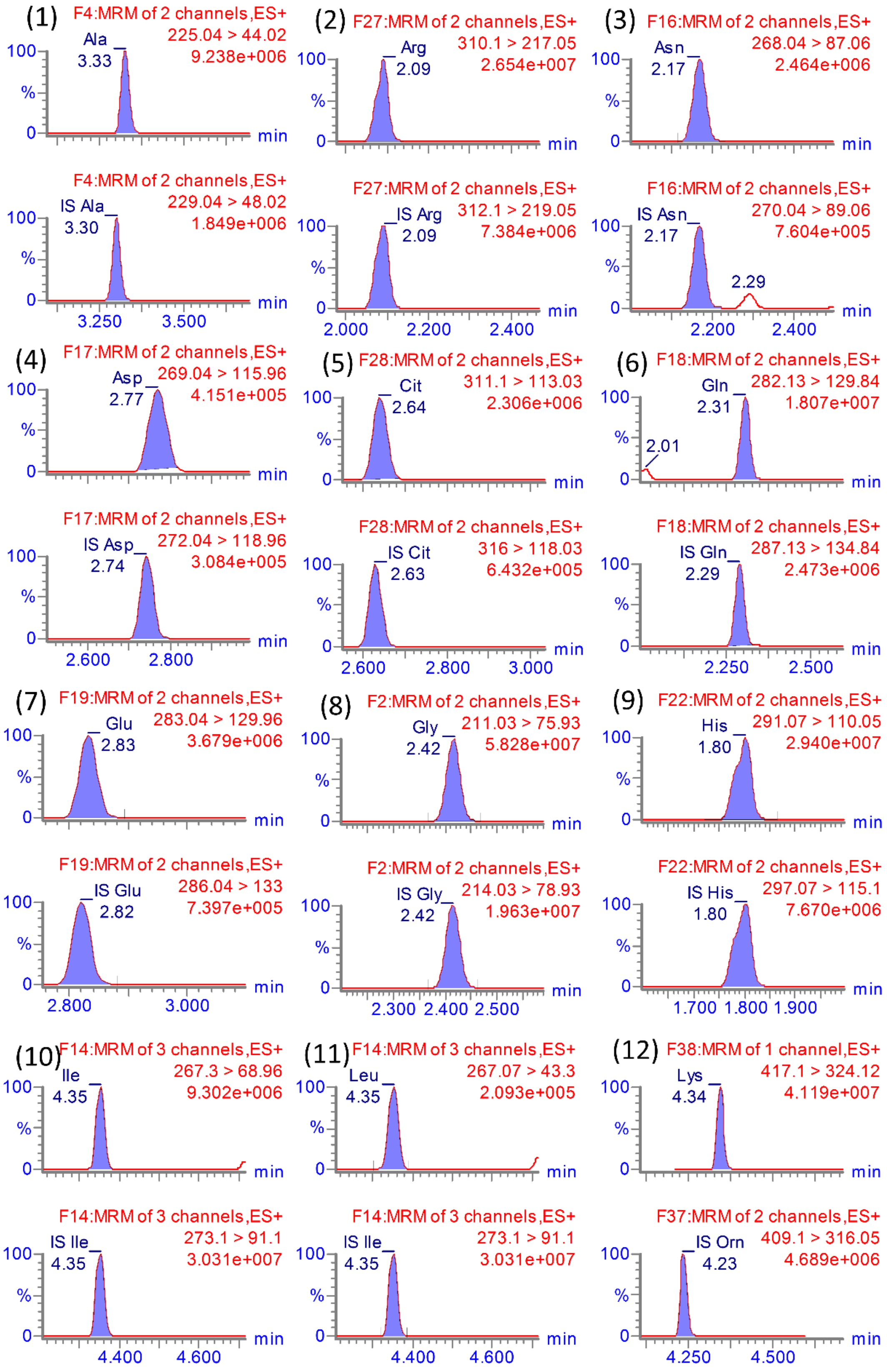

(1) F4:MRM of 2 channels,ES+
225.04 > 44.02
9.238e+006
Ala
3.33
F4:MRM of 2 channels,ES+
229.04 > 48.02
1.849e+006
IS Ala
3.30
3.250
3.500
(2) F27:MRM of 2 channels,ES+
310.1 > 217.05
2.654e+007
Arg
2.09
F27:MRM of 2 channels,ES+
312.1 > 219.05
7.384e+006
IS Arg
2.09
2.000
2.200
2.400
(3) F16:MRM of 2 channels,ES+
268.04 > 87.06
2.464e+006
Asn
2.17
F16:MRM of 2 channels,ES+
270.04 > 89.06
7.604e+005
IS Asn
2.17
2.29
2.200
2.400
(4) F17:MRM of 2 channels,ES+
269.04 > 115.96
4.151e+005
Asp
2.77
F17:MRM of 2 channels,ES+
272.04 > 118.96
3.084e+005
IS Asp
2.74
2.600
2.800
(5) F28:MRM of 2 channels,ES+
311.1 > 113.03
2.306e+006
Cit
2.64
F28:MRM of 2 channels,ES+
316 > 118.03
6.432e+005
IS Cit
2.63
2.600
2.800
3.000
(6) F18:MRM of 2 channels,ES+
282.13 > 129.84
1.807e+007
Gln
2.31
2.01
F18:MRM of 2 channels,ES+
287.13 > 134.84
2.473e+006
IS Gln
2.29
2.250
2.500
(7) F19:MRM of 2 channels,ES+
283.04 > 129.96
3.679e+006
Glu
2.83
F19:MRM of 2 channels,ES+
286.04 > 133
7.397e+005
IS Glu
2.82
2.800
3.000
(8) F2:MRM of 2 channels,ES+
211.03 > 75.93
5.828e+007
Gly
2.42
F2:MRM of 2 channels,ES+
214.03 > 78.93
1.963e+007
IS Gly
2.42
2.300 2.400 2.500
(9) F22:MRM of 2 channels,ES+
291.07 > 110.05
2.940e+007
His
1.80
F22:MRM of 2 channels,ES+
297.07 > 115.1
7.670e+006
IS His
1.80
1.700 1.800 1.900
(10) F14:MRM of 3 channels,ES+
267.3 > 68.96
9.302e+006
Ile
4.35
F14:MRM of 3 channels,ES+
273.1 > 91.1
3.031e+007
IS Ile
4.35
4.400
4.600
(11) F14:MRM of 3 channels,ES+
267.07 > 43.3
2.093e+005
Leu
4.35
F14:MRM of 3 channels,ES+
273.1 > 91.1
3.031e+007
IS Ile
4.35
4.400
4.600
(12) F38:MRM of 1 channel,ES+
417.1 > 324.12
4.119e+007
Lys
4.34
F37:MRM of 2 channels,ES+
409.1 > 316.05
4.689e+006
IS Orn
4.23
4.250
4.500
100
%
0
min

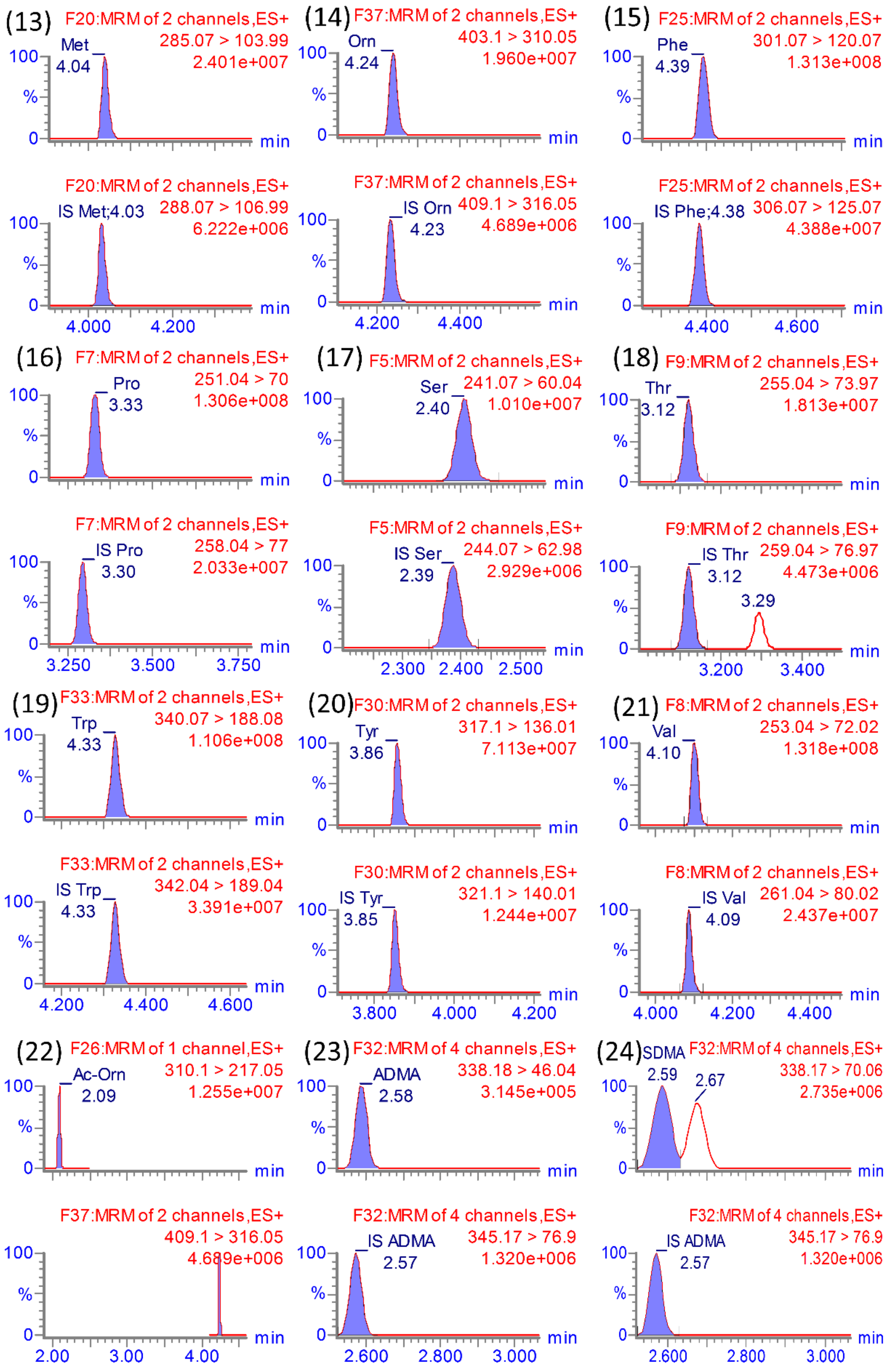
(13) F20:MRM of 2 channels,ES+
285.07 > 103.99
2.401e+007
Met
4.04
F20:MRM of 2 channels,ES+
288.07 > 106.99
6.222e+006
IS Met;4.03
4.000 4.200
(14) F37:MRM of 2 channels,ES+
403.1 > 310.05
1.960e+007
Orn
4.24
F37:MRM of 2 channels,ES+
409.1 > 316.05
4.689e+006
IS Orn
4.23
4.200 4.400
(15) F25:MRM of 2 channels,ES+
301.07 > 120.07
1.313e+008
Phe
4.39
F25:MRM of 2 channels,ES+
306.07 > 125.07
4.388e+007
IS Phe;4.38
4.400 4.600
(16) F7:MRM of 2 channels,ES+
251.04 > 70
1.306e+008
Pro
3.33
F7:MRM of 2 channels,ES+
258.04 > 77
2.033e+007
IS Pro
3.30
3.250 3.500 3.750
(17) F5:MRM of 2 channels,ES+
241.07 > 60.04
1.010e+007
Ser
2.40
F5:MRM of 2 channels,ES+
244.07 > 62.98
2.929e+006
IS Ser
2.39
2.300 2.400 2.500
(18) F9:MRM of 2 channels,ES+
255.04 > 73.97
1.813e+007
Thr
3.12
F9:MRM of 2 channels,ES+
259.04 > 76.97
4.473e+006
IS Thr
3.12
3.29
3.200 3.400
(19) F33:MRM of 2 channels,ES+
340.07 > 188.08
1.106e+008
Trp
4.33
F33:MRM of 2 channels,ES+
342.04 > 189.04
3.391e+007
IS Trp
4.33
4.200 4.400 4.600
(20) F30:MRM of 2 channels,ES+
317.1 > 136.01
7.113e+007
Tyr
3.86
F30:MRM of 2 channels,ES+
321.1 > 140.01
1.244e+007
IS Tyr
3.85
3.800 4.000 4.200
(21) F8:MRM of 2 channels,ES+
253.04 > 72.02
1.318e+008
Val
4.10
F8:MRM of 2 channels,ES+
261.04 > 80.02
2.437e+007
IS Val
4.09
4.000 4.200 4.400
(22) F26:MRM of 1 channel,ES+
310.1 > 217.05
1.255e+007
Ac-Orn
2.09
F37:MRM of 2 channels,ES+
409.1 > 316.05
4.689e+006
2.00 3.00 4.00
(23) F32:MRM of 4 channels,ES+
338.18 > 46.04
3.145e+005
ADMA
2.58
F32:MRM of 4 channels,ES+
345.17 > 76.9
1.320e+006
IS ADMA
2.57
2.600 2.800 3.000
(24) SDMA F32:MRM of 4 channels,ES+
338.17 > 70.06
2.735e+006
2.59
2.67
F32:MRM of 4 channels,ES+
345.17 > 76.9
1.320e+006
IS ADMA
2.57
2.600 2.800 3.000
100
%
0
min

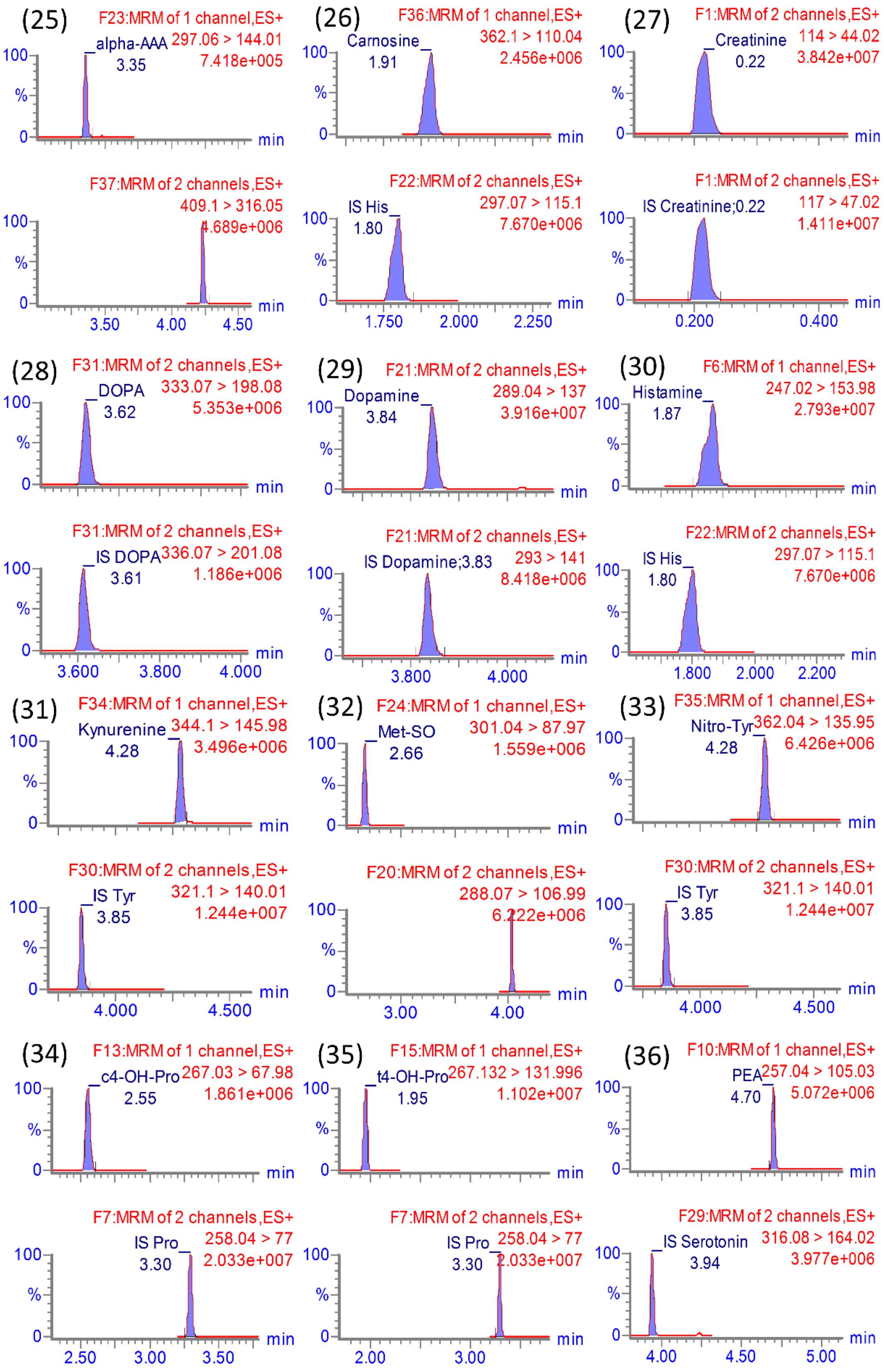
(25)
F23:MRM of 1 channel,ES+
alpha-AAA 3.35
297.06 > 144.01
7.418e+005
F37:MRM of 2 channels,ES+
409.1 > 316.05
4.689e+006
(26)
F36:MRM of 1 channel,ES+
Carnosine 1.91
362.1 > 110.04
2.456e+006
F22:MRM of 2 channels,ES+
IS His 1.80
297.07 > 115.1
7.670e+006
(27)
F1:MRM of 2 channels,ES+
Creatinine 0.22
114 > 44.02
3.842e+007
F1:MRM of 2 channels,ES+
IS Creatinine;0.22
117 > 47.02
1.411e+007
(28)
F31:MRM of 2 channels,ES+
DOPA 3.62
333.07 > 198.08
5.353e+006
F31:MRM of 2 channels,ES+
IS DOPA 3.61
336.07 > 201.08
1.186e+006
(29)
F21:MRM of 2 channels,ES+
Dopamine 3.84
289.04 > 137
3.916e+007
F21:MRM of 2 channels,ES+
IS Dopamine;3.83
293 > 141
8.418e+006
(30)
F6:MRM of 1 channel,ES+
Histamine 1.87
247.02 > 153.98
2.793e+007
F22:MRM of 2 channels,ES+
IS His 1.80
297.07 > 115.1
7.670e+006
(31)
F34:MRM of 1 channel,ES+
Kynurenine 4.28
344.1 > 145.98
3.496e+006
F30:MRM of 2 channels,ES+
IS Tyr 3.85
321.1 > 140.01
1.244e+007
(32)
F24:MRM of 1 channel,ES+
Met-SO 2.66
301.04 > 87.97
1.559e+006
F20:MRM of 2 channels,ES+
288.07 > 106.99
6.222e+006
(33)
F35:MRM of 1 channel,ES+
Nitro-Tyr 4.28
362.04 > 135.95
6.426e+006
F30:MRM of 2 channels,ES+
IS Tyr 3.85
321.1 > 140.01
1.244e+007
(34)
F13:MRM of 1 channel,ES+
c4-OH-Pro 2.55
267.03 > 67.98
1.861e+006
F7:MRM of 2 channels,ES+
IS Pro 3.30
258.04 > 77
2.033e+007
(35)
F15:MRM of 1 channel,ES+
t4-OH-Pro 1.95
267.132 > 131.996
1.102e+007
F7:MRM of 2 channels,ES+
IS Pro 3.30
258.04 > 77
2.033e+007
(36)
F10:MRM of 1 channel,ES+
PEA 4.70
257.04 > 105.03
5.072e+006
F29:MRM of 2 channels,ES+
IS Serotonin 3.94
316.08 > 164.02
3.977e+006
100
%
0
min

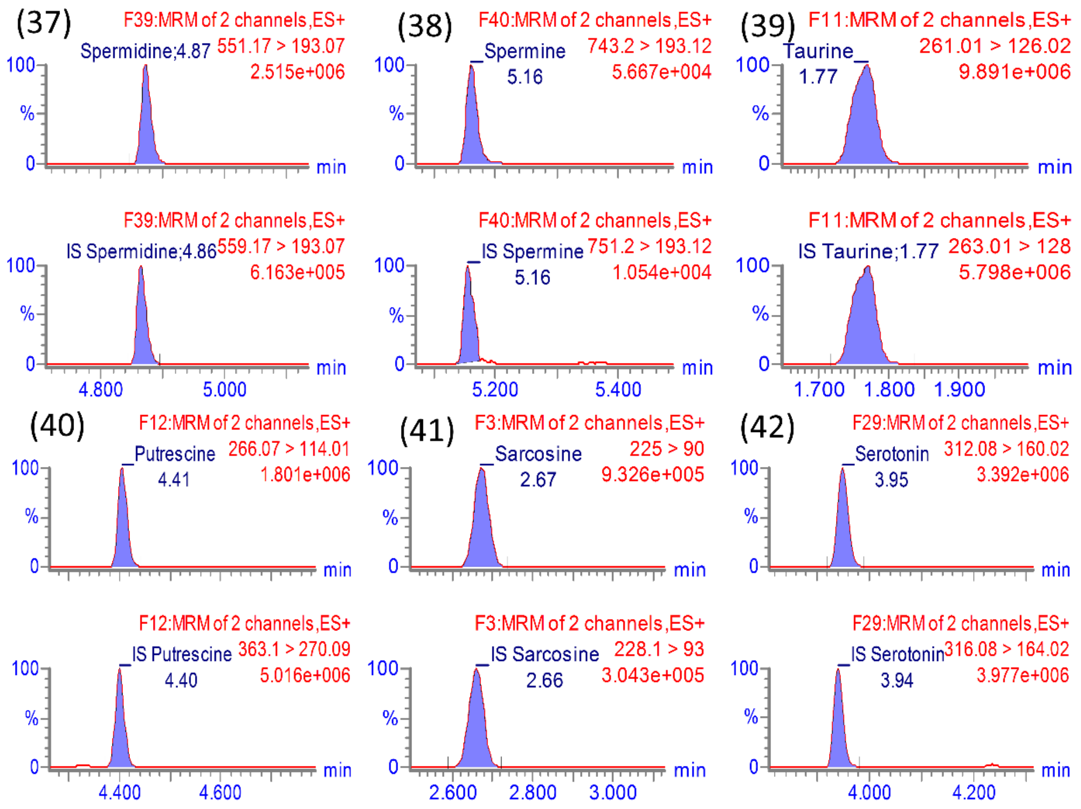

Figure S2. Chromatograms of standards and related internal standards from Liquid Chromatography-Tandem Mass Spectrometry (LC/MSMS) analysis.

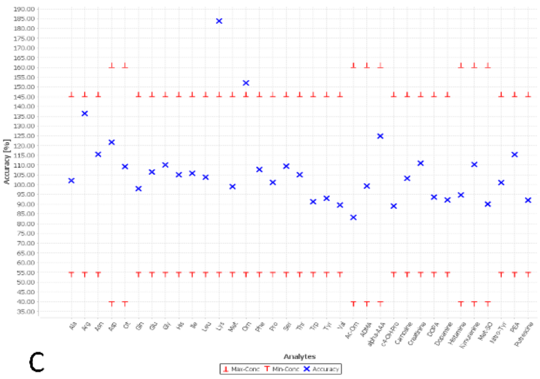

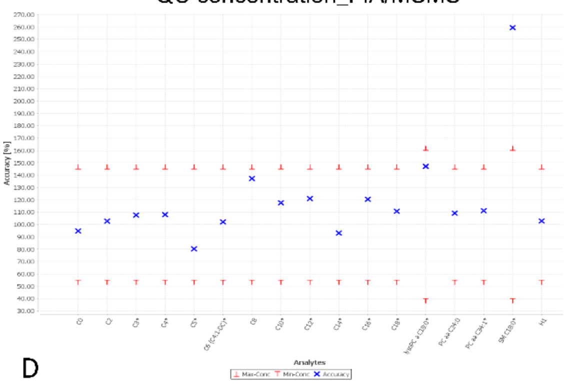

C

Analytes

	Name	Tolerance [%]	Accuracy [%]	Concentration [µM]	Expected Concentration [µM]	Measurement Status
1	Ala	45.000	102.079	773.601	757.845103	Valid
2	Arg	45.000	136.422	259.716	190.376797	Valid
3	Asn	45.000	115.525	166.390	144.029665	Valid
4	Asp	60.000	121.700	171.430	140.862279	Valid
5	Cit	60.000	109.250	169.979	155.587147	Valid
6	Gln	45.000	97.940	805.775	822.725764	Valid
7	Glu	45.000	106.471	376.335	353.462396	Valid
8	Gly	45.000	110.111	930.415	844.982124	Valid
9	His	45.000	105.053	200.848	191.188573	Valid
10	Ile	45.000	105.792	199.981	189.033390	Valid
11	Leu	45.000	103.806	223.427	215.235343	Valid
12	Lys	45.000	183.829	750.379	408.193812	STD/QC > Limit
13	Met	45.000	99.009	168.405	170.091515	Valid
14	Orn	45.000	152.096	267.987	176.195421	STD/QC > Limit
15	Phe	45.000	107.727	193.731	179.835062	Valid
16	Pro	45.000	101.121	373.958	369.811336	Valid
17	Ser	45.000	109.444	207.932	189.989841	Valid
18	Thr	45.000	105.051	195.157	185.773968	Valid

D

Analytes

	Name	Tolerance [%]	Accuracy [%]	Concentration [µM]	Expected Concentration [µM]	Measurement Status
1	C0	45.000	94.795	49.496	52.213798	Valid
2	C2	45.000	102.774	12.566	12.226394	Valid
3	C3*	45.000	107.659	4.908	4.558927	Valid
4	C4*	45.000	108.049	4.415	4.085809	Valid
5	C5*	45.000	80.314	3.661	4.558148	Valid
6	C6 (C4:1-DC)*	45.000	102.134	2.240	2.192991	Valid
7	C6 (C4:1-DC)*	45.000	102.134	2.240	2.192991	Valid
8	C8	45.000	137.290	2.913	2.122016	Valid
9	C10*	45.000	117.636	2.118	1.800746	Valid
10	C12*	45.000	121.031	4.729	3.907068	Valid
11	C14*	45.000	93.120	1.578	1.694066	Valid
12	C16*	45.000	120.477	4.328	3.592733	Valid
13	C18*	45.000	110.749	2.147	1.938894	Valid
14	lysoPC a C18...	60.000	147.129	124.151	84.382223	Valid
15	PC aa C24:0	45.000	109.223	22.645	20.732522	Valid
16	PC aa C34:1*	45.000	111.226	130.757	117.560000	Valid
17	SM C18:0*	60.000	259.511	53.200	20.500000	STD/QC > Limit
18	H1	45.000	102.885	13617.391	13235.609042	Valid

Figure S3. Concentration of metabolites in quality control (QC) with medium level. The concentration ratios (measured concentration/expected concentration) of quality control sample were from Liquid Chromatography-Tandem Mass Spectrometry (LC/MSMS) (**A**) and Flow Injection Analysis-Tandem Mass Spectrometry (FIA-MSMS) (**B**) The MetIQ software was used to automatically check whether the measured values of QC samples were within the ranges set. If the concentration ratio of the analyte was out of range, the analyte was marked yellow on the result table (**C**) (**D**).
